# Association between vitiligo and sexual dysfunction: current evidence

**DOI:** 10.1080/07853890.2023.2182906

**Published:** 2023-03-09

**Authors:** Xin Liang, Fei Guo, Xiaoce Cai, Jiao Wang, Jiale Chen, Li Liu, Yan Chen, Fang Liu, Yuhua Du, Lei Li, Xin Li

**Affiliations:** a Chinese medicine department, Songnan Town Community Health Service Center, Shanghai, China; b Department of Dermatology, Yueyang Hospital of Integrated Traditional Chinese and Western Medicine, Shanghai University of Traditional Chinese Medicine, Shanghai, China; c Institute of Dermatology, Shanghai Academy of Traditional Chinese Medicine, Shanghai, China

**Keywords:** Vitiligo, sexual dysfunction, ASEX, AVFSFI, meta-analysis

## Abstract

**Background:**

We discovered that vitiligo was associated with sexual dysfunction in clinical diagnosis and treatment; however, no further analysis had been performed due to a lack of data.

**Objective:**

This study aimed to clarify the relationship between vitiligo and sexual dysfunction.

**Methods:**

We searched six databases (PubMed, Embase, Cochrane, China National Knowledge Infrastructure, China Science and Technology Journal, and Wanfang Data Knowledge Service Platform) for nearly 40 years.

**Results:**

According to the search strategy, 91 relevant studies were retrieved, of which 4 were included in the analysis. The Arizona Sexual Experience Scale (ASEX) score (mean difference [MD] 4.96, 95% confidence interval [CI] 2.78–7.13, *p* < 0.00001) was higher in the vitiligo group than in the control group. The Arabic version of the Female Sexual Function Index (AVFSFI) score (mean difference [MD] − 3.40, 95% confidence interval [CI] − 5.49 to −1.31, *p* = 0.001) was lower in the vitiligo group than in the control group.

**Conclusions:**

Patients with vitiligo were found to be at greater risk of sexual dysfunction. Moreover, the association between vitiligo and sexual dysfunction was stronger in women than in men.

**Key Messages**
- Patients with vitiligo were found to be at greater risk of sexual dysfunction.
- The association between vitiligo and sexual dysfunction was stronger in women than in men.

## Introduction

Vitiligo is a commonly acquired pigmented disease characterized by the destruction of epidermal melanocytes (MC), which results in decreased melanin production and skin depigmentation. Although vitiligo affects approximately 1% of the global population, its prevalence does not vary significantly by sex, ethnicity, or geographic region [1]. Its pathogenesis has not been elucidated. However, research has shown that the etiology is closely related to genetics, autoimmune diseases, melanocyte self-destruction, trace element deficiency, environmental triggers, epidermal oxidative stress, and other factors [2–5]. The commonly used methods for vitiligo treatment in modern medicine include drug therapy (oral glucocorticoids, immunomodulatory trace elements, topical glucocorticoids, and calcineurin inhibitors), phototherapy (narrow-wave ultraviolet and 308 excimer laser), and surgical treatment (surgical skin grafting and depigmentation treatment) [6].

Vitiligo is generally asymptomatic because skin lesions can cause local or systemic depigmentation, which affects the appearance and greatly impacts the normal social activities and mental health of patients [7], especially in individuals with darker skin.

The skin has a role in sexual function, and skin damage can adversely affect sexual function [8]. More than 50% of patients with vitiligo experienced association problems with the opposite sex, and most felt that their sex lives had been immediately impacted [9]. Several studies have shown that vitiligo has an adverse impact on sexual relationship [8,10–12]. A recent systematic review showed that more than a quarter of patients had sexual dysfunction [13]. Some studies suggest that sexual relationship problems caused by vitiligo are more pronounced in men and single people [12], while other studies suggest that they are more pronounced in women [8].

There is currently a lack of meta-analyses on the relationship between vitiligo and sexual dysfunction; therefore, we searched all relevant clinical databases on vitiligo and sexual dysfunction and quantitatively analyzed their results. Furthermore, we analyzed the effect of sex differences on the relationship between vitiligo and sexual dysfunction.

## Materials and methods

### Data sources and searches

The PubMed, Embase, Cochrane Central Register of Controlled Trials, China Network Knowledge Infrastructure, China Science and Technology Journal Database, and Wan Fang Database were searched from inception to December 31, 2021, for literature on the relationship between vitiligo and sexual dysfunction using search keywords including vitiligo, sex relationship, sex dysfunction, and sex function.

### Study selection

**Figure 1.**
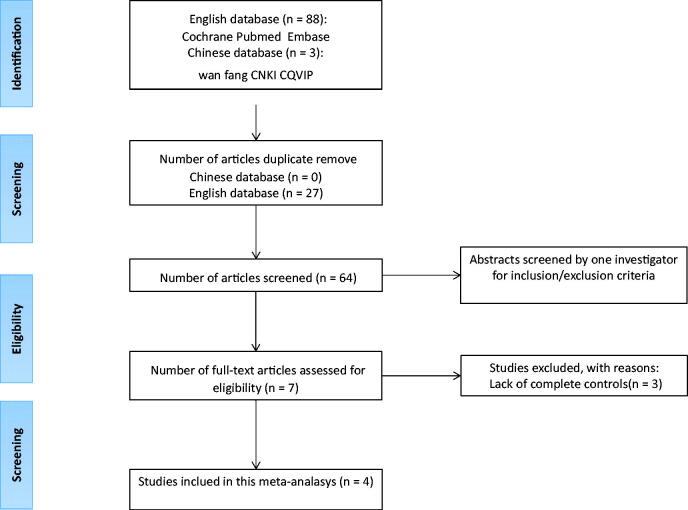
Literature search and study selection.

Publications were selected based on the following inclusion criteria for the studies: (1) human-only studies; (2) having a control group; (3) original data could be extracted; and (4) data were assessed using odds ratios (ORs), estimated confidence intervals (CIs) for hazard ratios, and hazard ratios (sufficient data could be used to calculate them), and sexual dysfunction was considered a specific outcome event. Initially, 91 articles were selected from the search (Figure 1). Then, these articles were carefully reviewed. Finally, this systematic review included four studies. A flowchart describing the screening process is shown in Figure 1.

### Data extraction

The following data were collected for each included study: (i) first author, (ii) study characteristics (year, duration, country, and design), (iii) participant characteristics (mean age, number of cases and controls, disease duration time, and quality of sexual life score), and (iv) outcome features.

### Quality assessment

Study quality was assessed using the Newcastle-Ottawa scale [14] (Table 1). This scale divides case-control studies into selection, comparable, and exposure categories and cohort studies into selection, comparable, and outcome categories. The selection category included four quality items, one for comparability and three for exposure.

**Table 1.** Newcastle–Ottawa Scale (NOS) quality assessment table.

Study	Selection	Comparability	Exposure/outcome	Overall star rating
Khaled et al. (2021) [15]	+++	++	++	7
Sarhan et al. (2016) [16]	+++	+	++	6
Sukan et al. (2007) [8]	+++	++	++	7
Yucel et al. (2021) [17]	+++	++	++	7

A star system was used to perform a semiquantitative assessment of the study quality. In the selection and exposure categories, a study adjudged a maximum of 4 stars for each numbered item. A maximum of 2 stars can be assigned for comparability. NOS ranges from 0 to 9 stars. We deemed high-quality studies that achieved ≥7 stars, medium-quality studies 4 to 6 stars, and poor-quality studies <4 stars.

### Data synthesis and analysis

Meta-analysis was performed using RevMan 5.4 software for Mac, and 95% CIs for mean differences (MD) and risk ratios (RR) were calculated for continuous and binary variables, respectively. If there was statistical heterogeneity (*p* < 0.10 or I^2^-values > 50%), a fixed-effects model was used; otherwise, a random-effects model was used. The methodology and results of this study strictly followed the meta-analysis of observational studies in epidemiology group guidelines and checklist [18].

## Results

Of the 91 relevant studies initially detected, 4 cross-sectional studies involving 365 participants with vitiligo aged 16–60 years were included (Figure 1). The characteristics of these studies are summarized in Table 2.

**Table 2.** Characteristics of included studies.

Author (pub. year)	Study setting	Study Period MM/ YY-MM/YY	Study design	Instruments used in the study	Measure of association (95%); mg/dL	Controls: total number	Cases: total number, number with GI, and number without GI (%)	Mean age of controls, years, mean (SD)	Mean age of cases, years, mean (SD)	Disease Duration, mean (SD)
Khaled et al. (2021) [15]	Egypt; NR	NR	case-control	AVFSFI	Control group: 26.6 (2.49) Vitiligo: 24.0 (3.84)	Total: 50	Total:50	35.7 (7.39)	37.3 (8.14)	NR
Sarhan et al. (2016) [16]	Egypt; outpatient	NR	Cross-sectional	AVFSFI	Control group: 26.1 (5.84) with GI: 19.82 (3.501) Without GI: 22.74 (5.246)	Total: 25	Total: 50 with GI: 25 Without GI: 25	31.7 (5.1)	with GI: 32.4 (7.5) Without GI: 30.3 (5.04)	With GI: 9.1 (5.3) Without GI: 5.2 (4.0)
Sukan et al. (2007) [8]	Turkey; outpatient	2/2003– 12/2004	Cross-sectional	ASEX	Female: Control group: 12.28 (4.03) Vitiligo: 18.42 (6.69) Male: Control group: 9.04 (3.36) Vitiligo: 10.65 (4.45)	Total: 50	Total: 50	35.98 (12.49)	35.82 (12.56)	NR
Yucel et al. (2021) [17]	Turkey; outpatient	11/2018– 5/2019	Cross-sectional	ASEX	Control group: 9.1 (3.3) with GI:15.0 (4.4) Without GI: 15.1 (4.8)	Total: 30	Total: 60 with GI: 30 Without GI: 30	37.53 (8.28)	with GI: 42.07 (9.82) Without GI: 40.17 (8.73)	With GI: 4 (3.73) Without GI: 1.75 (1,73)

AVFSFI: Arabic version of the Female Sexual Function Index; ASEX: Arizona Sexual Experience Scale; GI: genital involvement; NR: not reported.

## Primary Outcomes

### Arizona sexual experience scale (ASEX) score [19]

Four studies [8,15–17] used different evaluation metrics to assess sexual function. Using a random-effects model, combining the results of these trials showed a significant difference in the ASEX scores between the vitiligo and control groups (MD: 4.96; 95% CI: 2.78, 7.13; *p* < 0.00001; Table 3, Figure S1). For the female ASEX score, the meta-analysis showed a significant difference between the vitiligo and control groups (MD: 6.80; 95% CI: 4.93, 8.68; *p* < 0.00001; Table 3, Figure S1). For the male ASEX score, the meta-analysis showed a difference between the vitiligo and control groups (MD: 2.83; 95% CI: 0.44, 5.21; *p* = 0.02; Table 3, Figure S1).

**Table 3.** ASEX scores of the vitiligo and control groups with distinction between males and females.

Trials	Vitiligo		Control		MD [95% CI]	*P*-value
	Mean	SD	Mean	SD		
**ASEX**						
Sukan et al. (2007) [8]	14.3796	6.8182	10.66	4.0202	3.72 [1.53, 5.91]	
Yucel et al. (2021) [17]	15.05	4.5654	9.1	3.3	5.95 [4.30, 7/60]	
Meta-analysis (random, I^2^=61%)					4.96 [2.78, 7.13]	<0.00001
**Female ASEX**						
Sukan et al. (2007) [8]	18.42	6.69	12.28	4.03	6.14 [3.03, 9.25]	
Yucel et al. (2021) [17]	16.5857	4.3123	9.4	3.7	7.19 [4.83, 9.54]	
Meta-analysis (Fixed, I^2^=0%)					6.80 [4.93, 8.68]]	<0.00001
**Male ASEX**						
Sukan et al. (2007) [8]	10.65	4.45	9.04	3.36	1.61 [−0.55, 3.77]	
Yucel et al. (2021) [17]	12.842	4.0929	8.8	2.86	4.04 [1.88, 6.20]	
Meta-analysis (random, I^2^=59%)					2.83 [0.44, 5.21]	0.02

CI: confidence interval; ASEX: Arizona Sexual Experience Scale.

### Female sexual Function Index (AVFSFI) score

Two studies [15,16] assessed the participants’ Arabic version of the Female Sexual Function Index (AVFSFI) scores [20]. Meta-analysis showed that the AVFSFI score of the vitiligo group was significantly lower than that of the control group (MD: −0.73; 95% CI: −1.49, 0.03; *p* = 0.001; Table 4, Figure S2). Meta-analysis indicated no statistical differences in arousal (MD: −3.40; 95% CI: −5.49, −1.31; *p* = 0.06; Table 4, Figure S2), orgasm (MD: −0.65; 95% CI: −1.36, 0.05; *p* = 0.07; Table 4, Figure S2), and pain (MD: −0.13; 95% CI: −0.29, 0.03; *p* = 0.11; Table 4, Figure S2). There were statistical differences in desire (MD: −0.26; 95% CI: −0.48, −0.03; *p* = 0.03; Table 4, Figure S2), satisfaction (MD: −1.11; 95% CI: −1.49, −0.73; *p* < 0.00001; Table 4, Figure S2), and lubrication (MD: −0.47; 95% CI: −0.91, −0.03; *p* = 0.04; Table 4, Figure S2).

**Table 4.** AVFSFI score of the vitiligo and control groups and scores of each component.

Trials	Vitiligo		Control		MD [95% CI]	*P*-value
	Mean	SD	Mean	SD		
**AVFSFI**						
Khaled et al. (2021) [15]	24.00	3.84	26.60	2.49	−2.60 [−3.87, −1.33]	
Sarhan et al. (2016) [16]	21.28	4.6538	26.10	5.84	−4.82 [−7.45, −2.19]	
Meta-analysis (random, I^2^=55%)					−3.40 [−5.49, −1.31]	0.001
**Arousal**						
Khaled et al. (2021) [15]	4.07	0.78	4.44	0.52	−0.37 [−0.63, −0.11]	
Sarhan et al. (2016) [16]	3.46	0.6323	4.61	1.17	−0.15 [−1.64, −0.66]	
Meta-analysis (random, I^2^=55%)					−0.73 [−1.49, 0.03]	0.06
**Desire**						
Khaled et al. (2021) [15]	4.04	0.74	4.24	0.65	−0.20 [−0.47, 0.07]	
Sarhan et al. (2016) [16]	3.096	0.7508	3.48	0.917	−0.38 [−0.80, 0.03]	
Meta-analysis (fixed, I^2^=0%)					−0.26 [−0.48, −0.03]	0.03
**Lubrication**						
Khaled et al. (2021) [15]	4.89	0.58	5.17	0.72	−0.28 [−0.54, −0.02]	
Sarhan et al. (2016) [16]	4.062	0.8468	4.80	1.01	−0.74 [−1.20, −0.28]	
Meta-analysis (random, I^2^=66%)					−0.47 [−0.91, −0.03]	0.04
**Orgasm**						
Khaled et al. (2021) [15]	3.84	1.11	4.18	0.87	−0.34 [−0.73, 0.05]	
Sarhan et al. (2016) [16]	3.464	1.3917	4.53	1.36	−1.07 [−1.72, −0.41]	
Meta-analysis (random, I^2^=71%)					−0.65 [−1.36, 0.05]	0.07
**Satisfaction**						
Khaled et al. (2021) [15]	4.00	1.47	5.00	0.71	−1.00 [−1.45, −0.55]	
Sarhan et al. (2016) [16]	3.35	1.2796	4.72	1.566	−1.37 [−2.08, −0.66]	
Meta-analysis (fixed, I^2^=0%)					−1.11 [−1.49, −0.73]	<0.00001
**Pain**						
Khaled et al. (2021) [15]	3.28	0.36	3.40	0.51	−0.12 [−0.29, 0.05]	
Sarhan et al. (2016) [16]	3.77	0.9592	3.97	0.916	−0.20 [−0.65, 0.25]	
Meta-analysis (fixed, I^2^=0%)					−0.13 [−0.29, 0.03]	0.11

CI: confidence interval; AVFSFI: Arabic version of the Female Sexual Function Index.

## Secondary Outcomes

### Dermatological Life Quality Index (DLQI)

Two studies [16,17] calculated the effect of vitiligo on the Dermatological Life Quality Index (DLQI) scores. The meta-analysis of the DLQI scores in patients with and without genital involvement (GI) compared with controls revealed differences (GI: MD, 8.28; 95% CI: 1.05, 15.51; *p* = 0.02; without GI: MD: 6.76; 95% CI: 0.60, 12.91; *p* = 0.03; Table 5, Figure S3).

**Table 5.** DLQI score and marital duration of the vitiligo and the control groups; the vitiligo group distinguishes the presence or absence of genital involvement.

Trials	Vitiligo		Control		MD [95% CI]	*P*-value
	Mean	SD	Mean	SD		
**1. DLQI**						
** 1.1 Combine**						
** **Sarhan et al. (2016) [16]	13.00	2.828	2.12	1.30	10.88 [9.95, 11.81]	
** **Yucel et al. (2021) [17]	4.75	5.6218	0.70	1.40	4.05 [2.54, 5.56]	
** **Meta-analysis (random, I^2^=98%)					7.49 [0.80,1 4.18]	0.03
** 1.2 With GI**						
** **Sarhan et al. (2016) [16]	14.00	2.15	2.12	1.3	11.88 [10.90, 12.86]	
** **Yucel et al. (2021) [17]	5.2	6.8	0.7	1.4	4.50 [2.02, 6.98]	
** **Meta-analysis (random, I^2^=97%)					8.28 [1.05, 15.51]	0.02
** 1.3 Without GI**						
** **Sarhan et al. (2016) [16]	12.00	3.102	2.12	1.30	9.88 [8.56, 11.20]	
** **Yucel et al. (2021) [17]	4.3	4.2	0.70	1.40	3.60 [2.02, 5.18]	
** **Meta-analysis (random, I^2^=97%)					6.76 [0.60, 12.91]	0.03
**2. Marriage Time**						
** 2.1 Combine**						
** **Sarhan et al. (2016) [16]	10.45	6.6216	7.2	4.5	3.25 [0.70, 5.80]	
** **Yucel et al. (2021) [17]	17.25	11.162	10.37	6.59	6.88 [3.20, 10.56]	
** **Meta-analysis (random, I^2^=97%)					4.81 [1.29, 8.33]	0.007
** 2.2 With GI**						
** **Sarhan et al. (2016) [16]	13.9	6.6	7.2	4.5	6.70 [3.57, 9.83]	
** **Yucel et al. (2021) [17]	18.5	11.32	10.37	6.59	8.13 [3.44, 12.82]	
** **Meta-analysis (random, I^2^=97%)					7.14 [4.54, 9.74]	<0.00001
** 2.3 Without GI**						
** **Sarhan et al. (2016) [16]	7.00	4.60	7.20	4.50	−0.20 [−2.72, 2.32]	
** **Yucel et al. (2021) [17]	16.00	11.05	10.37	6.59	5.63 [1.03, 10.23]	
** **Meta-analysis (random, I^2^=97%)					2.38 [−3.29, 8.06]	0.41

CI: confidence interval; DLQI: Dermatological Life Quality Index; GI: genital involvement.

### Marriage time

Two studies [16,17] demonstrated a significant influence of marriage time between patients with vitiligo and controls (MD: 4.81; 95% CI: 1.29, 8.33; *p* = 0.007; Table 5, Figure S3). However, no significant difference was found between patients with vitiligo without genital involvement and controls using random-effects modeling of the same two studies [16,17] (MD: 2.38; 95% CI: −3.29, 8.06; *p* = 0.41; Table 5, Figure S3).

### Education

Two studies [8,17] analyzed the influence of vitiligo on the participants’ educational attainment. There were no significant differences in educational attainment between the vitiligo and control groups, including primary education (MD: 1.15; 95% CI: 0.75, 1.74; *p* = 0.53; Table 6, Figure S4) and high education (MD: 0.73; 95% CI: 0.38, 1.42; *p* = 0.36; Table 6, Figure S4).

**Table 6.** Educational attainment and fertility between the vitiligo and control groups.

Trials	Vitiligo		Control		RR [95% CI]	*P*-value
	Events	Total	Events	Total		
**1. Education**						
** 1.1 High education**						
** **Sukan et al. (2007) [8]	9	50	8	50	1.13 [0.47, 2.68]	
** **Yucel et al. (2021) [17]	17	60	15	30	0.57 [0.33, 0.97]	
** **Meta-analysis (fixed, I^2^=44%)					0.73 [0.38, 1.42]	0.36
** 1.2 Primary education**						
** **Sukan et al. (2007) [8]	41	50	42	50	0.98 [0.82, 1.17]	
** **Yucel et al. (2021) [17]	43	60	15	30	1.43 [0.97, 2.12]	
** **Meta-analysis (random, I^2^=74%)					1.15 [0.75, 1.74]	0.53
**2. Fertility**						
** 2.1 children 0**						
** **Sarhan et al. (2016) [16]	2	50	5	25	0.20 [0.04, 0.96]]	
** **Yucel et al. (2021) [17]	6	60	2	30	1.50 [0.32, 6.99]	
** **Meta-analysis (random, I^2^=69%)					0.55 [0.08, 3.97]	0.55
** 2.2 children 1**–**2**						
** **Sarhan et al. (2016) [16]	28	50	14	25	1.00 [0.65, 1.53]	
** **Yucel et al. (2021) [17]	24	60	21	30	0.57 [0.39, 0.84]]	
** **Meta-analysis (random, I^2^=72%)					0.75 [0.43, 1.30]	0.31
** 2.3 children 3**–**4**						
** **Sarhan et al. (2016) [16]	20	50	6	25	1.67 [0.77, 3.62]	
** **Yucel et al. (2021) [17]	30	60	7	30	2.14 [1.07, 4.30]	
** **Meta-analysis (fixed, I^2^=0%)					1.92 [1.14, 3.22]	0.01

CI: confidence interval.

### Fertility

Comparing patients with vitiligo with the controls, two studies [16,17] suggested that there was no significant discrepancy between those with one to two children (MD: 0.75; 95% CI: 0.43, 1.30; *p* = 0.31; Table 6, Figure S4) and those without children (MD: 0.55; 95% CI: 0.08, 3.97; *p* = 0.55; Table 6, Figure S4). A significant discrepancy was found in those with three to four children (MD: 1.92; 95% CI: 1.14, 3.22; *p* = 0.01; Table 6, Figure S4).

## Discussion

Vitiligo is a relatively common acquired pigmented skin disease that manifests as localized or generalized complete depigmentation of the skin and mucous membrane. It is caused by the disappearance of melanocyte function in the skin; however, the mechanism is unclear. Vitiligo varies in severity; the course of the disease is unpredictable and requires lifelong therapy [21]. Although vitiligo is not life-threatening or physically incapacitating, it can greatly affect a patient’s mental health [9,10,22], sexual dysfunction [8,12,15–17,23], and quality of life [11,16,22,23]. Since the 1980s, only seven studies [8,15–17,22–24] have investigated the possible association between vitiligo and sexual dysfunction. Some studies suggest that sexual relationship problems caused by vitiligo are more pronounced in men and single people [12], while others suggest that they are more pronounced in women [8]. Therefore, a meta-analysis study is needed to further examine the correlation between vitiligo and sexual dysfunction [12].

The outcomes of our study demonstrated that patients with vitiligo were at a greater risk of developing sexual dysfunction, and the association was stronger in female patients (Table 3, Figure S1). Sexual dysfunction in female patients may be more closely related to desire, lubrication, and satisfaction (Table 4, Figure S2). This problem may be due to the patient’s embarrassment, inferiority complex, and their partner’s embarrassment. Vitiligo is considered a disfiguring skin disease that places a heavy social and psychological burden. Sexual dysfunction is psychosocial comorbidity in more than a quarter of the patients with vitiligo, and female patients have a higher psychosocial burden [13].

Vitiligo is known to seriously impair patients’ quality of life [16,17,25–28]. Our results confirmed this: patients had higher DLQI scores, regardless of genital involvement (Table 5, Figure S3). This may be related to the implementation of DLQI scoring, which measures functional and lifestyle impairment from skin disease but not the emotional impact of the disease.

These results suggest that during the treatment of vitiligo, attention should be paid to patients’ quality of life and psychosocial health, and corresponding treatment strategies should be actively provided. Three studies [29–31] showed that in participants without vitiligo, sufficient knowledge of the disease promoted more positive interactions with people with vitiligo than in participants without the knowledge of the disease. Our results showed that compared with controls, patients with vitiligo had longer marital durations, and these were more pronounced in patients with genital involvement (Table 5, Figure S3). Therefore, if the knowledge of vitiligo disease is popularized among the family members and sexual partners of patients with vitiligo, along with psychological counseling, it may reduce the psychological burden of the patients themselves and improve their quality of life, sexual partners and families.

This meta-analysis revealed no significant difference in educational attainment between patients with vitiligo and healthy individuals (Table 6, Figure S4). This may indicate that vitiligo and educational attainment do not affect each other; however, further verification is needed. Although there was no significant difference in fertility between patients with vitiligo and controls, among participants, there were more patients with vitiligo with three to four children than the controls (Table 6, Figure S4). In the future, we may explore the relationship between vitiligo and fertility, given that no relevant literature has been reported.

## Limitations

This study had some limitations. First, the included studies were from Asia and Africa only, as data from studies in Europe and the United States are lacking. Second, this study was highly heterogeneous. Third, the previous systematic literature review [13] of the psychosocial effects of vitiligo, including sexual dysfunction, was qualitative, whereas this study was quantitative. Regarding the data analysis, ASEX and AVFSFI were the main judging indicators in our study, while the previous study used ASEX only [13]. Our article described the relationship between vitiligo and sexual dysfunction, showing that women with vitiligo are more likely to have sexual dysfunction than men. The previous study did not describe this topic in detail [13].

## Conclusions

In conclusion, patients with vitiligo are at greater risk of sexual dysfunction. The association between vitiligo and sexual dysfunction was stronger in women than in men. Physicians should be aware of this potential risk and actively intervene to reduce comorbidities.

## Supplementary Material

Supplemental Material

## Data Availability

All data generated or analyzed during this study are included in this published article (and its supplementary information files).

## References

[1] Liu W, Liu X-Y, Qian Y-T, et al. Urinary metabolomic investigations in vitiligo patients. Sci Rep. 2020;10(1):17989. 33093609 10.1038/s41598-020-75135-0 PMC7582886

[2] Alkhateeb A, Fain PR, Thody A, et al. Epidemiology of vitiligo and associated autoimmune diseases in caucasian probands and their families. Pigment Cell Res. 2003;16(3):208–214. 12753387 10.1034/j.1600-0749.2003.00032.x

[3] Spritz RA. The genetics of vitiligo. J Invest Dermatol. 2011;131(E1):E18–E20. 22094401 10.1038/skinbio.2011.7 PMC3513341

[4] Laddha NC, Dwivedi M, Mansuri MS, et al. Vitiligo: interplay between oxidative stress and immune system. Exp Dermatol. 2013;22(4):245–250. 23425123 10.1111/exd.12103

[5] Rodrigues M, Ezzedine K, Hamzavi I, et al. New discoveries in the pathogenesis and classification of vitiligo. J Am Acad Dermatol. 2017;77(1):1–13. 10.1016/j.jaad.2016.10.048 28619550

[6] Ezzedine K, Eleftheriadou V, Whitton M, et al. Vitiligo. Lancet. 2015;386(9988):74–84. 25596811 10.1016/S0140-6736(14)60763-7

[7] Trmoss and cjstevenson Incidence of male genital vitiligo. Br J Vener Dis. 1981;57:145–146. 7214123 10.1136/sti.57.2.145 PMC1045893

[8] Sukan M, Maner F. The problems in sexual functions of vitiligo and chronic urticaria patients. Journal of Sex & Marital Therapy. 2007;33(1):55–64. 17162488 10.1080/00926230600998482

[9] Porter JR, Beuf AH, Lerner AB, et al. The effect of vitiligo on sexual relationships. J Am Acad Dermatol. 1990;22(2 Pt 1):221–222. 2312803 10.1016/0190-9622(90)70028-g

[10] Porter J, Beuf A, Nordlund JJ, et al. Personal responses of patients to vitiligo: the importance of the patient-physician interaction. Arch Dermatol. 1978;114(9):1384–1385. 686757

[11] Papadopoulos L, Bor R, Legg C, et al. Impact of life events on the onset of vitiligo in adults: preliminary evidence for a psychological dimension in aetiology. Clin Exp Dermatol. 1998;23(6):243–248. 10233617 10.1046/j.1365-2230.1998.00384.x

[12] Parsad D, Dogra S, Kanwar AJ. Quality of life in patients with vitiligo. Health Qual Life Outcomes. 2003;1:58. 14613564 10.1186/1477-7525-1-58 PMC269995

[13] Ezzedine K, Eleftheriadou V, Jones H, et al. Psychosocial effects of vitiligo: a systematic literature review. Am J Clin Dermatol. 2021;22(6):757–774. 34554406 10.1007/s40257-021-00631-6 PMC8566637

[14] Stang A. Critical evaluation of the newcastle–ottawa scale for the assessment of the quality of nonrandomized studies in meta-analyses. Eur J Epidemiol. 2010;25(9):603–605. 20652370 10.1007/s10654-010-9491-z

[15] Khaled H, et al. Female sexual dysfunction in patients with psoriasis and vitiligo: an egyptian pilot study. J Egypt Women Dermatol Soc. 2021;18(1):22–34.

[16] Sarhan D, Mohammed GFA, Gomaa AHA, et al. Female genital dialogues: female genital Self-Image, sexual dysfunction, and quality of life in patients with vitiligo with and without genital affection. J Sex Marital Ther. 2016;42(3):267–276. 25650731 10.1080/0092623X.2015.1010678

[17] Yucel D, Sener S, Turkmen D, et al. Evaluation of the dermatological life quality index, sexual dysfunction and other psychiatric diseases in patients diagnosed with vitiligo with and without genital involvement. Clin Exp Dermatol. 2021;46(4):669–674. 33191544 10.1111/ced.14511

[18] Stroup DF, Berlin JA, Morton SC, et al. Meta-analysis of observational studies in epidemiology: a proposal for reporting. Meta-analysis of observational studies in epidemiology (MOOSE) group. Jama. 2000;283(15):2008–2012. 10789670 10.1001/jama.283.15.2008

[19] Mcgahuey AC, Gelenberg AJ, Laukes CA, et al. The Arizona sexual experience scale (ASEX): reliability and validity. Journal of Sex & Marital Therapy. 2000;26:25–40. 10693114 10.1080/009262300278623

[20] Anis TH, Gheit SA, Saied HS, et al. Arabic translation of female sexual function index and validation in an egyptian population. J Sex Med. 2011;8(12):3370–3378. 21995610 10.1111/j.1743-6109.2011.02471.x

[21] Lu T, Gao T, Wang A, et al. Vitiligo prevalence study in shaanxi province, China. Int J Dermatol. 2007;46(1):47e–451. 17214719 10.1111/j.1365-4632.2006.02848.x

[22] Borimnejad L, Parsa Yekta Z, Nikbakht-Nasrabadi A, et al. Quality of life with vitiligo: comparison of male and female muslim patients in Iran. Gender Medicine. 2006;3(2):124–130. 16860271 10.1016/s1550-8579(06)80201-9

[23] Alhetheli GI. The impact of vitiligo on patients’ psychological status and sexual function: cross-sectional questionnaire-based study. TODJ. 2021;15(1):23–30.

[24] Wang K-Y, Wang K-H, Zhang Z-P, et al. Health-related quality of life and marital quality of vitiligo patients in China. J Eur Acad Dermatol Venereol. 2011;25(4):429–435. 20666878 10.1111/j.1468-3083.2010.03808.x

[25] Mechri A, Amri M, Douarika AA, et al. J. Psychiatric mor- bidity and quality of life in vitiligo: a case controlled study [in french]. Tunis Med. 2006;84(10):632–635. 17193855

[26] Ongenae K, Dierckxsens L, Brochez L, et al. Quality of life and stigmatization profile in a cohort of vitiligo patients and effect of the use of camouflage. Dermatology. 2005;210(4):279–285. 15942213 10.1159/000084751

[27] Ongenae K, Van Geel N, De Schepper S, et al. Effect of vitiligo on self- reported health-related quality of life. Br J Dermatol. 2005;152(6):1165–1172. 15948977 10.1111/j.1365-2133.2005.06456.x

[28] Prćić S, Durović D, Duran V, et al. Some psychological characteristics of children and adolescents with vitiligo: our results [in serbian]. Med Pregl. 2006;59(5-6):265–269. 17039911 10.2298/mpns0606265p

[29] Tsadik AG, Teklemedhin MZ, Mehari Atey T, et al. Public knowledge and attitudes towards vitiligo: a sur- vey in mekelle city, Northern Ethiopia. Dermatol Res Pract. 2020;2020:1–7. 10.1155/2020/3495165 PMC7281839 32549889

[30] Fatani MI, Aldhahri RM, Otaibi A, et al. Acknowledging popular misconceptions about vitiligo in Western Saudi Arabia. J Dermatol Dermatol Surg. 2016;20(1):27–31.

[31] Juntongjin P, Rachawong C, Nuallaong W. Knowledge and attitudes towards vitiligo in the general population: a survey based on the simulation video of a real situation. Dermatol Sin. 2018;36(2):75–78.

