# Supplementary material for: Association between vitiligo and sexual dysfunction: current evidence
Source: Ann Med. 2023 Mar 9;55(1):946–53. doi: 10.1080/07853890.2023.2182906 (PMC10795651; doi:10.1080/07853890.2023.2182906)
Supplement: Supplemental Material [file IANN_A_2182906_SM2593.pptx]

## Slide 1
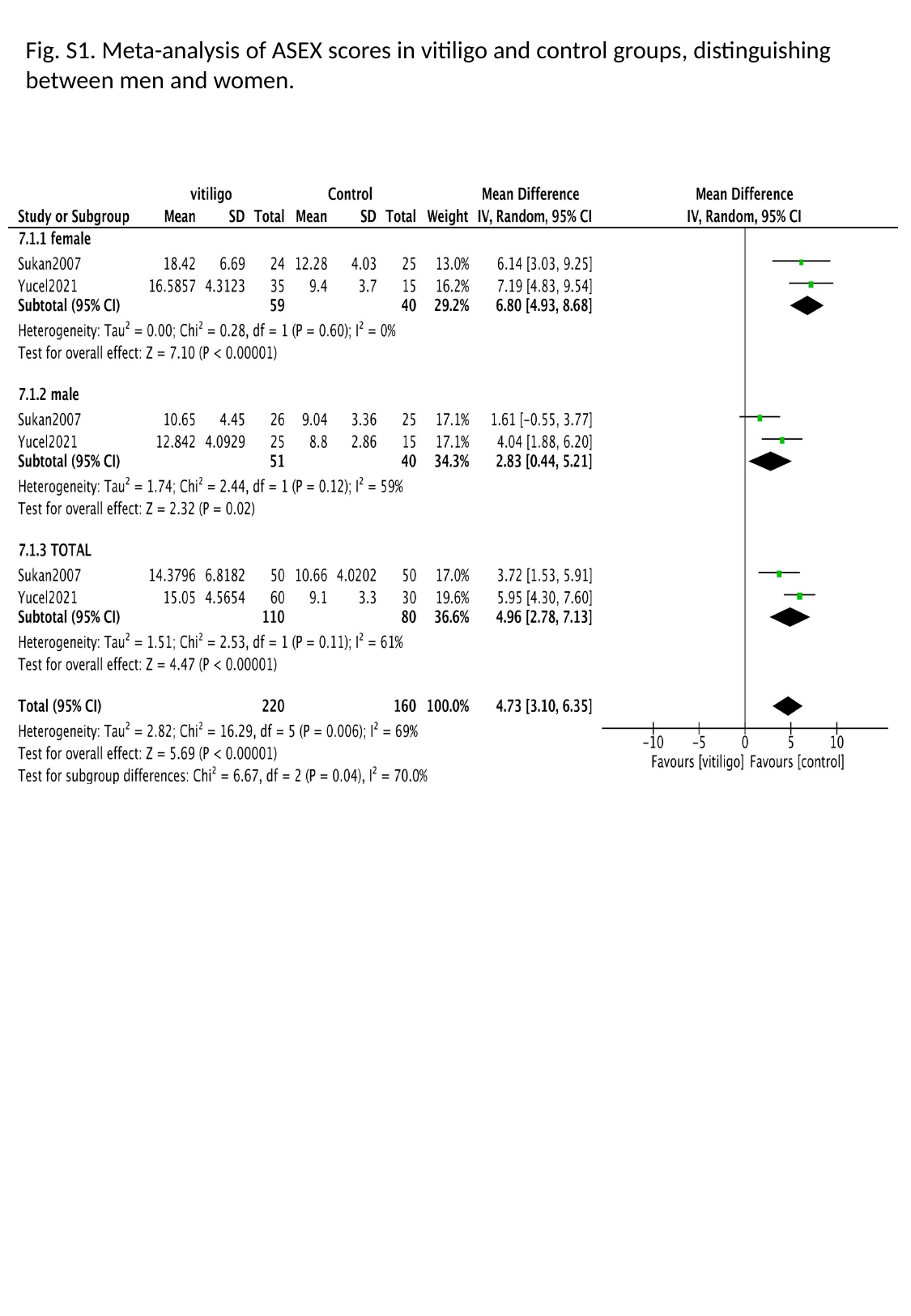

Fig. S1. Meta-analysis of ASEX scores in vitiligo and control groups, distinguishing between men and women.

## Slide 2
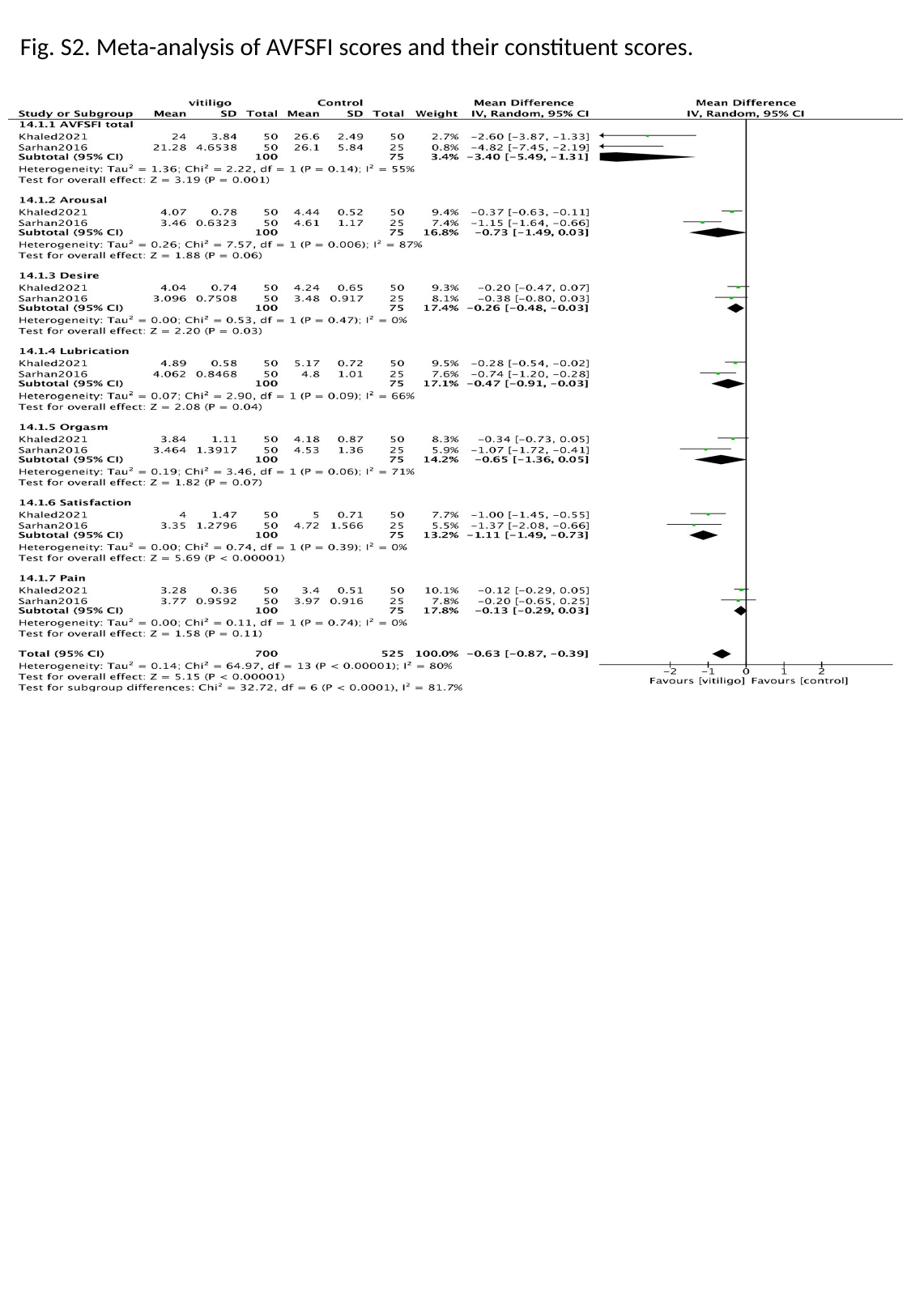

Fig. S2. Meta-analysis of AVFSFI scores and their constituent scores.
#

## Slide 3
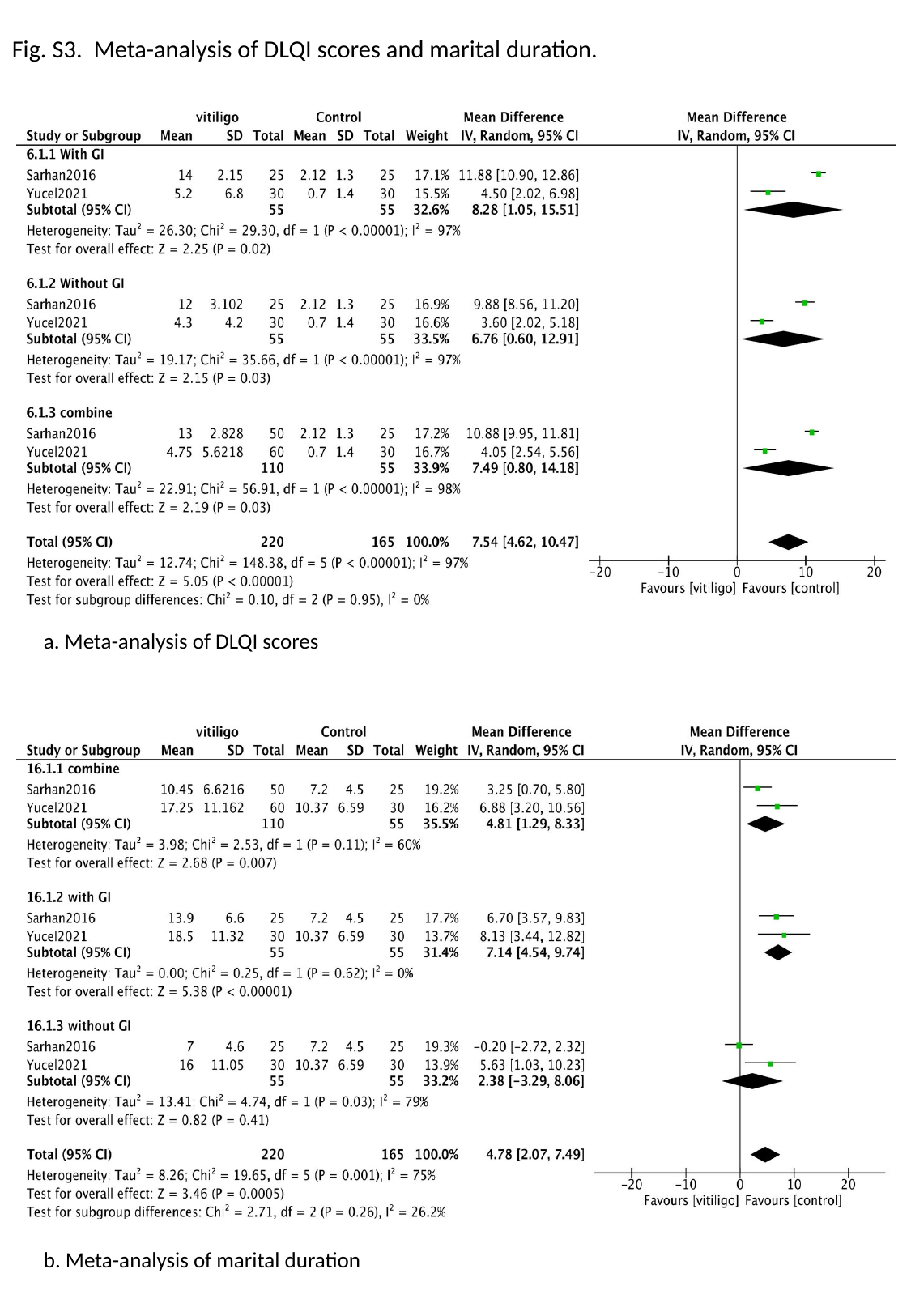

Fig. S3. Meta-analysis of DLQI scores and marital duration.
a. Meta-analysis of DLQI scores
b. Meta-analysis of marital duration

## Slide 4
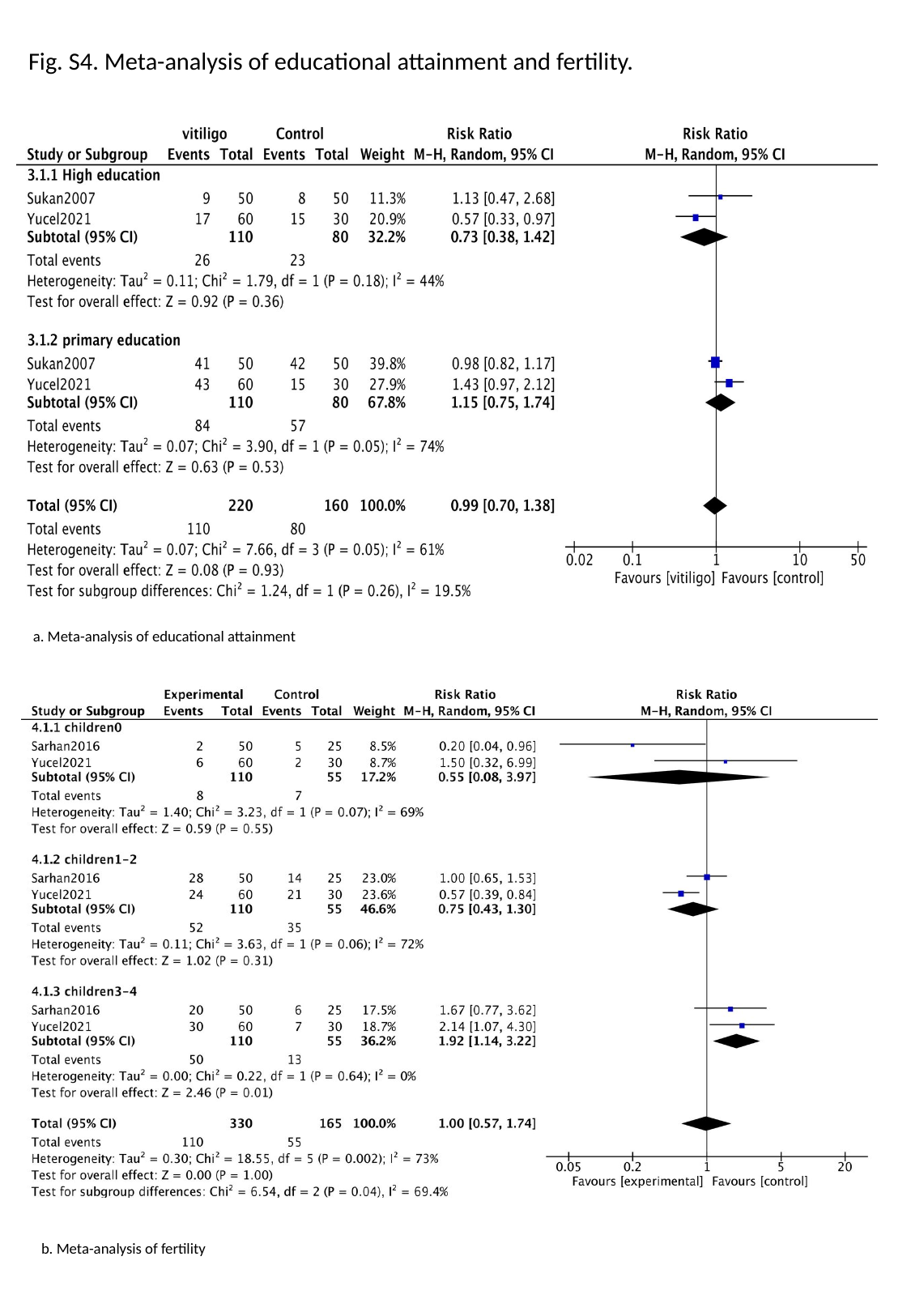

Fig. S4. Meta-analysis of educational attainment and fertility.
a. Meta-analysis of educational attainment
b. Meta-analysis of fertility
